# Diffusion Tensor Imaging in Alzheimer’s Disease and Mild Cognitive Impairment

**DOI:** 10.3233/BEN-2009-0234

**Published:** 2009-10-21

**Authors:** G. T. Stebbins, C. M. Murphy

**Affiliations:** Department of Neurological SciencesRush University Medical CenterChicagoILUSA

**Keywords:** Dementia, fractional anisotropy, mean diffusivity, perforant path, tractography, entorhinal cortex, magnetic resonance imaging, hippocampus, memory, medial temporal lobe

## Abstract

Structural magnetic resonance imaging (MRI) studies of Alzheimer’s disease and mild cognitive impairment (MCI) have focused on the hippocampus and entorhinal cortex; gray matter structures in the medial temporal lobe. Few studies have investigated the integrity of white matter in patients with AD or MCI. Diffusion tensor imaging (DTI) is a MRI technique that allows for the interrogation of the microstructural integrity of white matter. Based on increases in translational diffusion (mean diffusivity: MD) and decreases directional diffusion (fractional anisotropy: FA) damage to white matter can be assessed. Studies have identified regions of increased MD and decreased FA in patients with AD and MCI in all lobes of the brain, as well as medial temporal lobe structures including the hippocampus, entorhinal cortex and parahippocampal white matter. The pattern of white matter integrity disruption tends to follow an anterior to posterior gradient with greater damage noted in posterior regions in AD and MCI. Recent studies have exploited inter-voxel directional similarities to develop models of white matter pathways, and have used these models to assess the integrity of inter-cerebral connections. Particular focus has been applied to the parahippocampal white matter (including the perforant path) and the posterior cingulum. Although many studies have found DTI indicators of impaired white matter in AD and MCI, other studies have failed to detect any differences in MD or FA between the groups, demonstrating the need for large replicative studies. DTI is an evolving technique and advances in its application ought to provide new insights into AD and MCI.

## 1. Introduction

Memory decline is one of the most common cognitive complaints in the elderly. During the course of aging, memory decline in some older persons may ultimately develop into a degenerative dementia such as Alzheimer's disease (AD). AD is the most common cause of dementia in the elderly and characterized by widespread cortical changes, loss of neurons, and presence of senile plaques and neurofibrillary tangles that are found in the medial temporal structures early in the course of the disease [16,17]. The presence of these pathological markers of AD is associated with a profound impairment of episodic memory, with a more variable pattern of additional cognitive and other deficits [56,79,94].

Non-demented individual with memory complaints or mild memory impairment may represent a transitional state between healthy aging and AD. It has been demonstrated that individuals with memory complaints perform poorly on episodic memory tests even though they do not meet criteria for dementia [1,33]. Additionally, individuals with amnestic mild cognitive impairment (MCI) convert to probable AD at an increased rate compared to older adults without memory problems [33,34,61,83] and decline in episodic memory performance at a faster rate than healthy aging, but less rapidly than individuals diagnosed with mild AD [12].

Because of the known relationship of medial temporal lobe structures and episodic memory processing [73] and because of the profound declarative memory impairment associated with AD and MCI, these structures have been a major focus of MRI investiga-

ISSN 0953-4180/09/$17.00 © 2009 – IOS Press and the authors. All rights reserved tions. Structural MRI studies have documented hippocampal and entorhinal atrophy in patients with AD, individuals with MCI and even in individuals with cognitive complaints but no objective memory testing impairments [21–23,25,47,53,54].

## 2. White matter damage in AD/MCI and diffusion tensor imaging

In addition to the examination of the hippocampi and entorhinal cortices in AD and MCI, there is increased interest in white matter changes in these conditions. Reports of pathological white matter changes have been documented in at least 50% of patients with AD [18]. Pathological white matter changes include decreased myelin density [74], decreased myelin basic protein [88], loss of oligodendrocytes [75] and microgila activation [37]. It has been suggested that the loss of oligodendrocytes and myelin damage in AD is due to increased vulnerability of later myelinating regions [5, 6,65]. This theory, termed retrogenesis [65], posits a reverse order to white matter degeneration. Earlier myelinating regions with larger diameter axons and a higher oligodendrocyte-to-axon ratio, such as primary motor and sensory cortical areas, are relatively spared in AD and not affected until late in the disease course. Later myelinating regions with smaller diameter axons and lower oligodendrocyte-to-axon ratios, such as the medial temporal lobe and other neocortical regions, are affected early in the course of the disease.

White matter damage leads to increases in brain water content, and because MRI signal is based on excitation and relaxation of hydrogen atoms, there are multiple MRI techniques to measure changes in water content. The MRI T2 signal decay rate is particularly sensitive to water content and has been used to document increased white matter damage in patients with AD [4]. Although T2 weighted MRI scanning is sensitive to white matter damage, it does not provide information on the microstructural integrity of white matter. One novel technique that takes advantage of this hydrogen-based alteration in MRI signal at the microstructural level is diffusion tensor imaging (DTI).

DTI is based on sensitizing the MR signal to movement of hydrogen on the order of several microns through the application of diffusion weighted gradients in at least six non-collinear gradients simultaneously, and measuring the direction and magnitude of hydrogen movement [7]. The application of at least six non-collinear gradients allows for examination of diffusion characteristics irrespective of head position. The three-dimensional geometry of the diffusion in a particular volume element (voxel) can be described by a mathematical construct called a “tensor” [8] that can be represented by a $3 \times 3$ matrix. From the diffusion tensor in each voxel, one can derive three eigenvalues ($\lambda_1$, $\lambda_2$ and $\lambda_3$) defining the magnitude of the diffusion system and the three associated eigenvectors that describe the direction of the diffusion system. The average of the three eigenvalues represents the mean molecular motion (mean diffusivity: MD) that is affected by barriers to diffusion, but does not provide information on the directionality of the diffusion. Based on the ratio of the three eigenvalues, the intra-voxel direction of hydrogen diffusion can be determined. This scalar measure is termed fractional anisotropy (FA), and can range from 0 to 1 [8], with 0 indicating completely random diffusion (isotropic diffusion) and 1 representing completely directional diffusion (anisotropic diffusion). CSF has extremely low FA values because hydrogen is free to diffuse in any direction. Gray matter has low FA because cellular structures (e.g., cell membrane, organelles) impede the free diffusion of hydrogen, but these structures do not promote organized, directional diffusion. Highly organized white matter tracts have high FA because hydrogen diffusion is directionally constrained by the tract’s cellular organization.

There is some evidence that changes in the individual eigenvalues of the tensor can provide information about the specifics of white matter damage. The primary eigenvalue represent the longitudinal direction of diffusion, or axial diffusion. The secondary and tertiary eigenvalues represent the transverse direction of diffusion, or radial diffusion. As axial diffusion decreases and radial diffusion increases, the shape of diffusion becomes more spherical. As axial diffusion increases and radial diffusion decreases, the shape of diffusion becomes more prolate. Decreased axial diffusion has been associated with axonal damage in mouse models [78], perhaps reflecting increased barriers to organized diffusion in the axial plane. Increased radial diffusion has been associated with damage to myelin [77], perhaps reflecting increased diffusion in the plane orthogonal to the axial plane.

The inter-voxel continuation of the primary eigenvalue can be used to develop models of white matter tracts, a technique termed tractography. Since, in white matter, the primary eigenvalue represents the primary direction of fiber orientation, continuation of similar eigenvalues across voxels represents the inter-voxel direction of the fiber pathway. As the similarity of eigenvectors continues across many voxels, the white matter tract is modeled [9,57]. These models can be used to define intra-cerebral connection, and measuring the average FA and MD of the tracts can infer the integrity of these various connections.

When the barriers to free diffusion of hydrogen in white matter degenerate, such as seen the white matter damage, mean diffusivity increases and the direction of intra-voxel diffusion becomes more isotropic. Thus, one would predict increased mean diffusivity and isotropic diffusion (decreased FA) in the white matter of patients with AD because of the degeneration of myelinated tracts due to damage to myelin and oligodendrocytes [74,75,88]. However, these expectations are rather simplistic, given the complexity of white matter architecture. In regions with a complex matrix of white matter organization, with crossing or other non-parallel fiber structures, DTI indicators of damage may manifest a pattern other than increased MD and decreased FA. For example, if damage occurs to a white matter region with an organizationally complex matrix with crossing fibers, increases in MD would occur due to increased translational diffusion. FA, however, would increase due to a decrease in crossing fibers or other non-parallel organization [81].

Alterations to the individual eigenvectors could also be variable in examinations of white matter changes in AD or MCI. As noted above, there is evidence of myelin damage in patients with AD [74,75,88]. Such damage would be expected to result in an increase in radial diffusion but leave axial diffusion intact [78,79]. However, one might also expect to find decreases in axial diffusion in AD because of Wallerian degeneration of white matter tracts resulting from loss of gray matter [62]. If both myelin damage and Wallerian degeneration are present in AD or MCI, decreases in both axial and radial diffusion would be evident. These same interpretative complexities are applicable to DTI changes in tractographic models of cerebral connections associated with memory function.

## 3. Methodological approaches to DTI studies of AD and MCI

Various methods of extracting DTI data have been employed in the study of white matter changes in AD and MCI. Typically, group comparisons are made from DTI scans of memory impaired individuals and healthy control participants. The two major approaches to analyzing these data are region-of-interest (ROI) and whole brain voxelwise analyses. ROI approaches construct either hand drawn ROIs on individual scans, or template-based ROIs applied to scans that have been warped into a common coordinate system to compensate for individual differences in brain morphology; a process called normalization. Whole-brain voxelwise methods always require the warping of individual scans into a common coordinate space and then interrogation of group differences proceeds on a voxel-by-voxel basis.

Each of these approaches has different strengths and weaknesses [76]. ROIs drawn on native images provide superior delineation of cerebral structures. The operator has great control over which voxels to include in the ROI. However, the drawing of multiple ROIs for many subjects can be prohibitively labor-intensive. Additionally, if more than one operator is constructing the ROIs, inter-operator reliability needs to be established. The use of clearly delineated landmarks can help increase the reliable construction of ROIs (e.g., [36]). If, instead of hand-drawn ROI of specific structure, a ROI of predetermined size and shape (e.g., a circle of 5 mm diameter) is used, the anatomical localization specificity is diminished, but processing time is facilitated.

Template-based ROIs applied to normalized scans have the advantage of being extremely time efficient, and large datasets can be processed quickly. Because the scans have been warped into a common coordinate system, only one ROI needs to be constructed that can then be applied to the entire sample. One concern with this approach is the accuracy of the normalization process. Because of individual differences in brain morphology, the alignment of different brain regions across individuals is not perfect. Another concern is the effect warping may have on the DTI scalar measures [48]. Additionally, because of the imperfect matching of individual brain structure, and the non-Gaussian nature of MRI voxelwise signal, the normalized volumes need to be adjusted by application of a smoothing kernel [49]. Often, a Gaussian full-width-at-half-maximum (FWHM) smoothing algorithm is applied to the data across a specified search region, although smoothing algorithms other than Gaussian have been proposed specifically for application with DTI data (e.g., [84]). The effect of this smoothing is to modify extreme values within the search region to promote a normal distribution of scores.

Both ROI approaches, in either native space or normalized space, are also subject to the effects of partial voluming. The DTI values extracted from the ROIs are averaged, in some fashion, for the entire ROI. If part of

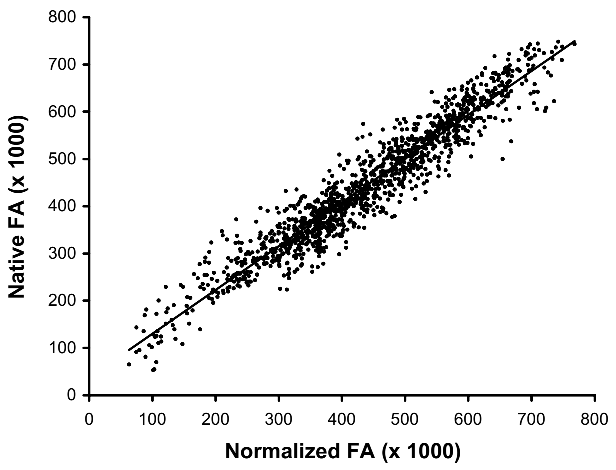

Fig. 1. Scatter plot of fractional anisotropy (FA) values from a normal control subject in normalized space (x-axis) and native space (y-axis). Normalization was achieved by application of 12 parameter affine transformation followed by an elastic (non-linear) deformation to achieve a closer match to the template image. The inverse transformation was used to convert normalized space back to native space. FA values were extracted from a $10 \times 10 \times 10$ voxel cube in both normalized and native volumes.

the ROI does not contain the tissue of interest, the resultant average will be a combination of both intended and unintended values. This potential confound is particularly applicable to ROIs used on normalized scans. If a template-based ROI or a ROI of pre-determined shape and size is applied to a region of potential differential atrophy, the problem of partial voluming is compounded. For example, if one uses a template-based ROI of the hippocampal region to study FA or MD differences between patients with AD or MCI and healthy controls, the effects of partial voluming would be greater in the memory impaired group because of the increased atrophy in that region. Thus the resultant values extracted from the ROI in the AD or MCI group would be derived from both tissue and CSF, whereas the values extracted from the normal group would be derived from non-atrophied tissue. This would result in lower FA values and higher MD values for the AD or MCI group, simply because of atrophy, and may not reflect the integrity of the remaining white matter in the ROI.

Whole-brain voxelwise approaches have the advantage of being extremely time efficient, so large numbers of scans can be processed quickly increasing the potential statistical power of analyses. Additionally, there are no a priori constraints of specific regions of analysis, so the search for differences proceeds in an unbiased manner. Potential weaknesses to this approach include the potential for mismatching between the native and normalized image because of imperfect warping algorithms [48], alteration of the DTI values due to the effects of normalization, and possible alteration of the DTI values due to smoothing. We examined the effects of normalization and smoothing on FA by comparing the normalized values to the native values on a voxel-by-voxel basis [58]. We found a high concordance between normalized and native voxelwise FA values resulting in an $R^2$ value of 0.93 (Fig. 1), suggesting little distortion introduced during the normalization process. However, smoothing with a 8 mm FWHM Gaussian filter did introduce larger discrepancies between smoothed and non-smoothed voxelwise FA values ($R^2 = 0.78$) (Fig. 2).

## 4. Studies using diffusion weighted imaging

It is possible to use diffusion weighted imaging to develop indices of anisotropic diffusion without developing a full tensor model. Instead of applying gradients in at least six non-collinear directions simultaneously, the gradients are applies sequentially. This allows for generation of a measure of diffusion direction, but this measure is susceptible to artifacts due to differences in head position during each gradient application. Studies using this approach to diffusion imaging have reported changes in both MD and anisotropic diffusion in patients with AD and MCI.

Altered diffusivity, as measured by diffusion weighted imaging in patents with AD and MCI, has been reported in many different regions. Increases in MD have been reported in patients with AD in the temporal lobes [40,51], hippocampus proper [51,70], splenium of the corpus callosum [38,70], posterior cingu-

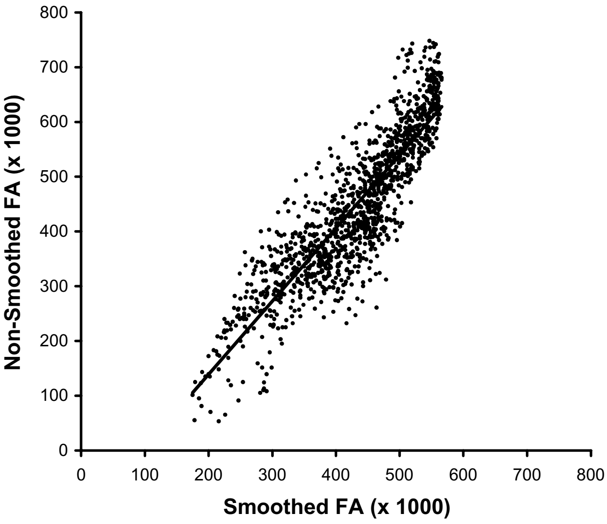

Fig. 2. Scatter plot of fractional anisotropy (FA) values from a normal control subject smoothed with a 8 mm full-width-half-maximum Gaussian kernel (x-axis) and non-smoothed (y-axis). Smoothed and non-smoothed FA values were extracted from a 10 × 10 × 10 voxel cube. Note distortion (shifting of extreme values towards the mean) at high and low values of FA.

lum, occipital lobes and parietal lobes [51]. Increases in isotropic diffusion in AD have been reported in the corpus callosum (particularly the splenium) [38] and temporal lobe [40]. Another study, however, reported no changes in MD or anisotropic diffusion direction in patients with mild to moderate AD [13]. In participants with MCI, increased hippocampal [51,64], temporal lobe and corpus callosal MD [64], and increased occipital lobe isotropic diffusion [51] has been reported.

A general interpretation of these results is that diffusion changes in AD and MCI tend to occur in more posterior regions as opposed to anterior regions, as seen in normal aging [41,42]. Such increased posterior isotropic diffusion was found in a sample of AD patients [70], and differentiated AD from a sample of patients with vascular dementia [39].

## 5. Diffusion tensor imaging in AD and MCI

The number of studies employing DTI to investigate white matter integrity in AD and MCI has greatly increased over time. The majority of these studies use DTI measures of MD and FA as markers of cerebral integrity, although the decomposition of individual eigenvalues to axial and radial diffusivity is becoming more common. Some studies use a whole-brain voxelwise analytic approach, although the majority of studies use ROIs to interrogate the status of cerebral integrity. There has been a recent increase in investigations of known or suspected memory system targets through the use of tractography or other indices of cerebral pathways.

### *5.1. Mean diffusivity in AD and MCI*

MD provides a measure of translational diffusion, and increases in the presence of tissue damage. This measure does not provide information about the directionality of diffusion. In AD, MD could be expected to increase in regions with tissue damage. Previous results have documented increased MD in most lobar regions of patient with AD, including frontal lobes [14,69], temporal lobes [14,15,32,41,69,80], parietal lobes [41, 69,92] and occipital lobe [41,69]. Increased MD has been reported in tracts and structures involved in intercerebral communications, including the parietal region of the superior longitudinal fasciculus [52], the corpus callosum [14] and the cingulum [31,55,60,92]. Hippocampal and parahippocampal increases in MD have also been reported [32,52,92]. Finally, MD increase has shown a significant negative correlation with a cognitive performance measures [59], particularly MD in the posterior cingulum [91].

The regional distribution of increased MD in AD seems to basically follow the pathology of AD, with many studies reporting this DTI indicator of tissue disruption in the hippocampus, temporal lobe and posterior cingulum. Other regions of increased MD in AD

tend to be found in more posterior regions. However, there are a number of studies that have failed to find increased MD in these regions in AD. For example, no significant increase in MD was reported in ROI investigations of the frontal lobe [41,80], parietal lobe [80], occipital lobe [14,15,32,80], corpus callosum [32,41, 52,92] and posterior cingulum [30,52].

The pattern of increased MD in individuals with MCI is similar to that seen in patients with AD, but not as extensive. Increases in MD have been reported in the frontal lobes [68], temporal lobe [32] including the hippocampus [32] and entorhinal cortex [68], parietal lobe [80], occipital lobe [32,68], and posterior cingulum [31,92]. As with the studies of MD in AD, there are studies that do not find increased MD in individuals with MCI in some or all of these regions (e.g., [30, 55,80]). Additionally, there are a few studies demonstrating an increase in MD in patients with AD, but no increase in individuals with MCI [30,80], suggesting a lesser pathological burden in the MCI patients.

### *5.2. Fractional anisotropic diffusion in AD and MCI*

FA provides a measure of the directionality of diffusion. In white matter, high FA is seen in highly organized tissue with parallel structure. Damage to white matter breaks down the organized structure leading to a decrease in FA. As such, measures of FA are thought to provide an *in vivo* marker of cerebral integrity. In AD and MCI, alterations in FA have been reported in multiple regions. For example, decreases in FA have been reported in the frontal lobes in some studies [14, 43,55,68,69], but not by others [32,41,80,85]. Conflicting findings of normal and decreased FA are also reported for the parietal lobes [14,32,43,55,69,80] with most studies finding no difference between AD, MCI and healthy controls. Most studies also report no differences in FA in the occipital lobes in AD, MCI or healthy controls [32,41,43,80].

Despite these conflicting reports of FA in AD, MCI and healthy controls, there are three regions that seem relatively consistent in findings of decreased FA. These regions recapitulate the development of pathological changes noted in AD [16,17], and include sub-regions of the medial temporal lobe including the hippocampus, entorhinal cortex and parahippocampal white matter [19,32,68,69,93], temporal lobes proper [14,32,43, 55,69,85,90], and the posterior cingulum [19,30,31,55, 60,68,85,92,93]. There is some evidence that the loss of integrity, as measured by DTI, is more pronounced in individuals with AD compared to MCI. This is seen in regions were patients with AD demonstrated significant decreases in FA compared to healthy controls, while patients with MCI do not. Such a dissociation of FA between AD and MCI has been noted in the temporal lobes and hippocampus [32] and the posterior cingulum [19], and suggest that the pathological changes altering FA in these regions in AD may not yet affect individuals with MCI.

### *5.3. Investigations of targeted regions involved in memory processing*

Medial temporal lobe structures, including the entorhinal cortex, hippocampus and perforant path, are essential for memory function and are known to be pathologically affected in AD [16,17]. The entorhinal cortex receives input from multiple cortical regions and transmits this input to the hippocampus [2,45,86] via the perforant path. Disconnection between the entorhinal cortex and hippocampus because of damage to the perforant path has been suggests as a possible contribution to the memory impairment found in AD and MCI [45].

DTI examinations of the medial temporal lobe region have focused on the white matter of the parahippocampal region. White matter fibers in this area not only provide intrinsic connections between the entorhinal cortex and the hippocampus through the perforant pathway, but also connections between the entorhinal cortex/hippocampus and the rest of the brain through the posterior cingulum. To examine this area, two basic approaches have been used. The first involves the creation of individual ROIs, usually drawn on high-resolution T1-weighted images that include only the white matter of interest. The second approach takes advantage of tractographic DTI techniques to define the white matter pathway and use this definition to extract information as to the integrity of the tract.

A study by Kalus and colleagues used a ROI approach to examine inter-voxel coherence of diffusion direction in the region of the perforant path [50]. In a group of 10 AD, 10 MCI and 10 healthy elderly controls, individual ROIs were constructed to encompass the hippocampus, entorhinal cortex and the specific parahippocampal region that includes the perforant path. These ROIs were used to extract a measure of inter-voxel coherence from those regions and compare the coherence between the groups. The authors found a significant decrease in inter-voxel coherence in the AD group in all three regions. Decreased coherence was found in the MCI group only in the parahippocampal white matter including the perforant path. Another study [66] used very similar methods to examine FA and MD in parahippocampal white matter in the region of the perforant path in MCI and healthy controls. The authors found that there was a significant increase in MD in this region in MCI, but no associated decrease in FA. Unfortunately, because of the different measures used by these two studies (inter-voxel coherence versus FA and MD), a direct comparison is not possible.

A different approach to studying parahippocampal white matter is seen in investigations that use tractography methods to model the pathway of white matter through this region. Salat et al. [69] used a tract-based technique (http://www.fmrib.ox.ac.uk/fsl/) to develop skeletonalized models of FA and MD in white matter pathways of patients with AD and healthy controls. By isolating the parahippocampal white matter they examined group differences in FA, radial diffusivity and axial diffusivity. The authors found significant decreases in FA in this region in the patients with AD compared to the healthy controls. The decrease was greater in the anterior portion of this pathway; a region associated with the perforant path. Additionally, they found a dissociation between radial diffusivity and axial diffusivity, such that radial diffusivity was significantly increased in AD, suggesting damage to the myelin in that region [77]. However, axial diffusivity was also *increased* in the AD group. Since decreases in axial diffusion have been associated with axonal damage [78], an increase in this measure in AD is not easy to interpret.

Tract-based analyses of the parahippocampal pathway have demonstrated indices of damage extending beyond the parahippocampal region and into the posterior cingulum. Nakata et al. [59,60] used tractography to develop a pathway model of the posterior cingulum. The tract model extended from the posterior cingulum to the central cingulum. Values of FA and MD were extracted from the developed tract and compared between the groups. Significant increases in MD and decreases in FA for found in the AD sample compared to healthy controls. Additionally there was a significant correlation between extracted MD values and a measure of mental status in the AD sample.

In another study, Zhou et al [93] used tract-based techniques to examine pathways connecting the hippocampal region to the posterior cingulum, hippocampal region to the whole brain, and posterior cingulum to the whole brain in a sample of AD, MCI and healthy controls. They found a significant reduction in the number of modeled fibers in hippocampus – whole brain pathway, hippocampus – posterior cingulum pathway and posterior cingulum – whole brain pathway in the AD sample. The MCI sample evidenced a significant reduction in the number of fibers in the hippocampus to whole brain pathway only. The authors suggested that these findings may provide a measure of disease severity and assist in the differentiation of AD and MCI.

Tract-based examinations of DTI derived indices of white matter integrity in AD and MCI have investigated pathways other than the parahippocampal and posterior cingulum. Fujie et al. [35] found that FA in the uncinate fasciculus, a pathway that connects the frontal and temporal lobes, was significantly reduced in MCI compared to healthy controls and that FA in this fasciculus in MCI was significantly correlated with memory performance. Stricker el al. [82] examined the integrity of early – and late-myelinating pathways in patients with AD. They found a significant reduction in FA in the late-myelinating pathways in the AD sample, but no difference in the early-myelinating pathways. This finding provides some support to the theory of retrogenesis [65] that posits relative sparing of early-myelinating regions in AD, but a higher susceptibility to damage in the late-myelinating regions [5,6].

## 6. Summary

DTI is an MRI scanning technique that allows for the examination of white matter microstructural integrity based on the directionality of diffusion in the brain. Two measures are most commonly reported: FA and MD. FA provides a measure of the directionality of diffusion and MD provides a measure of translational diffusion. In intact tissue, MD in constrained by barriers to free diffusion and FA is determined by the parallel organization of the tissue. In white matter, directional diffusion is promoted along the long axis of the axons and perpendicular diffusion is impeded. Damage to white matter results in an increase in MD through the loss of barriers to free diffusion, and FA is decreased by a loss of barriers to perpendicular diffusion.

DTI indices of cerebral damage are commonly found in AD and MCI. The majority of DTI changes appear to be in more posterior regions [41,55] compared to frontal regional changes that are more common in healthy aging [42], however, many studies have found increased MD and decreased FA in multiple locations in the brains of patients with AD and MCI. The most commonly reported regions of DTI alterations are the temporal lobes, with particular emphasis on the parahippocampal white matter, and the posterior cingulum.

Both of these regions are strongly implicated in memory function, and DTI indices of damage may help identify the pathological substrates to AD and MCI. The severity of alterations in MD and FA appears to be greater in AD compared to MCI, with individuals with MCI typically evidencing fewer regions of altered DTI values. There is some evidence that changes in MD are more typical in MCI whereas as changes in MD and FA are more typical in AD. Additionally, there is some evidence that changes in radial diffusivity, a potential marker of myelin damage, is more common in AD and MCI than changes in axial diffusivity, an indicator of axonal damage [43,69]. Further study will be needed to address this issue.

An important consideration in the utility DTI in the study of AD and MCI is not only its sensitivity to group differences, but also its sensitivity to assess the association of the DTI metrics to cognitive performance measures. In many studies, MD and FA show a significant association with cognitive functions typically impaired in AD and MCI, including mental status (e.g., [30, 31,35,50,59,66,91,93]) suggesting external validity of these measures because of their relationship to cognitive performance and group differences.

In is important to note that there are many conflicting reports on regional disruption of DTI measures in AD and MCI compared to healthy controls. Indeed, there are a few studies that do not find any significant differences in DTI measures between memory-impaired subjects and controls [41,52]. The reasons for these conflicting reports are not clear. Most studies are conducted with small samples sizes, so statistical power is decreased and differences in disease characteristics (e.g., level of cognitive impairment) may contribute to increased variability. The use of ROI versus whole-brain voxelwise versus tract-based methodologies may provide differential sensitivities to group differences in DTI measures. Finally, differences in scanning parameters such as voxel sizes, may introduce differences in partial voluming, and thus cause differential measurement error between studies.

Techniques such as tract-based analysis of white matter pathways, whole-brain voxelwise approaches and individually traced ROIs hold promise for providing additional information on the status of white matter in AD and MCI. Advances in DTI pulse sequences, including increased resolution, parallel imaging and development of sequences that are increasingly immune to distortions introduced by fast imaging will also improve our ability to detect differences in white matter integrity in patients with AD and MCI. The use of multiple imaging modalities to assess both functional changes in AD and MCI along with measures of connective networks through tract-based DTI analyses will advance our understanding of effects of dementia on gray matter, white matter, and their interaction.

## Acknowledgments

This work was supported by grant P01 AG09466 from the National Institute on Aging, National Institutes of Health.
